# Respiratory viral infections in pragmatically selected adults in intensive care units

**DOI:** 10.1038/s41598-021-99608-y

**Published:** 2021-10-08

**Authors:** Cong-Tat Cia, I-Ting Lin, Jen-Chieh Lee, Huey-Pin Tsai, Jen-Ren Wang, Wen-Chien Ko

**Affiliations:** 1 Division of Critical Care Medicine, Department of Internal Medicine, National Cheng Kung University Hospital, College of Medicine, National Cheng Kung University, Tainan, Taiwan; 2 Center for Infection Control, National Cheng Kung University Hospital, College of Medicine, National Cheng Kung University, Tainan, Taiwan; 3 Department of Pathology, National Cheng Kung University Hospital, College of Medicine, National Cheng Kung University, Tainan, Taiwan; 4 Department of Medical Laboratory Science and Biotechnology, National Cheng Kung University, Tainan, Taiwan; 5 Center of Infectious Disease and Signaling Research, National Cheng Kung University, Tainan, Taiwan; 6 National Institute of Infectious Diseases and Vaccinology, National Health Research Institutes, Tainan, Taiwan; 7 Division of Infectious Diseases, Department of Internal Medicine, National Cheng Kung University Hospital, College of Medicine, National Cheng Kung University, No. 138, Sheng Li Road, Tainan, 704 Taiwan; 8 Department of Medicine, College of Medicine, National Cheng Kung University, Tainan, Taiwan

**Keywords:** Clinical microbiology, Infectious-disease diagnostics, Influenza virus, Viral infection

## Abstract

Respiratory viruses can be detected in 18.3 to 48.9% of critically ill adults with severe respiratory tract infections (RTIs). The present study aims to assess the clinical significance of respiratory viruses in pragmatically selected adults in medical intensive care unit patients and to identify factors associated with viral respiratory viral tract infections (VRTIs). We conducted a prospective study on critically ill adults with suspected RTIs without recognized respiratory pathogens. Viral cultures with monoclonal antibody identification, in-house real-time polymerase chain reaction (PCR) for influenza virus, and FilmArray respiratory panel were used to detect viral pathogens. Multivariable logistic regression was applied to identify factors associated with VRTIs. Sixty-four (40.5%) of the included 158 critically ill adults had respiratory viruses detected in their respiratory specimens. The commonly detected viruses included influenza virus (20), followed by human rhinovirus/enterovirus (11), respiratory syncitial virus (9), human metapneumovirus (9), human parainfluenza viruses (8), human adenovirus (7), and human coronaviruses (2). The FilmArray respiratory panel detected respiratory viruses in 54 (34.6%) patients, but showed negative results for seven of 13 patients with influenza A/H3 infection. In the multivariable logistic regression model, patient characters associated with VRTIs included those aged < 65 years, household contact with individuals with upper RTI, the presence of fever, cough with sputum production, and sore throat. Respiratory viruses were not uncommonly detected in the pragmatically selected adults with critical illness. The application of multiplex PCR testing for respiratory viruses in selected patient population is a practical strategy, and the viral detection rate could be further improved by the patient characters recognized in this study.

Respiratory viruses can cause upper and lower respiratory tract infections (RTIs) including seasonal colds, otitis media, bronchiolitis, pneumonia, and acute deterioration of chronic lung diseases, in patients with and without suppressed immunity^1,2^. Viral pathogens may cause 24.5% of cases of community-acquired pneumonia requiring hospitalization in adults, as modern multiplex molecular diagnostic assays are applied^3,4^. Moreover, up to 10% of community-acquired pneumonia is concurrently caused by viral and bacterial pathogens^3^.

Respiratory viruses had been detected in respiratory specimens in 18.3% of critically ill adults requiring invasive mechanical ventilation^5^. The detection rates are higher, ranging from 20.5 to 49.0%, in patients with community or hospital-acquired lower respiratory tract infections admitted to intensive care units (ICUs)^5–14^. Human rhinovirus, influenza virus, and human parainfluenza viruses (HPIVs) are the most frequently detected viruses^5–9^. In terms of disease severities requiring timely actions, critically ill patients are considered appropriate candidates for respiratory viral panel testing^15^.

As the expense of the molecular tests remains high, universal testing for all patients with suspected RTIs may not be a cost-effective strategy, since more than half events are caused by bacteria or other non-infectious etiologies^3^. Proper selection for patients to test is required to maximize the clinical benefits in medical care, either by early initiation of antiviral agents, avoidance of unnecessary invasive studies, or shorter antibiotic exposure^15^. Patients with known bacterial pathogens may be less likely to benefit from testing for respiratory viruses, except for potentially treatable influenza virus, since discontinuation of antimicrobial therapy is not a reasonable option for them. However, a patient with viral but no bacterial infection may not benefit from antimicrobial agents but experience only adverse effects, which were noted in 20% of hospitalized patients^16^. Combined with negative microbiological tests for bacterial infections, a confirmed viral RTI (VRTI) can support the clinical decision to discontinue or de-escalate antibacterial therapies. The current study aims to assess the clinical significance of respiratory viruses in pragmatically selected adults in medical ICUs and to delineate clinical variables associated with viral RTIs (VRTIs).

## Methods

### Study design and population

We conducted a prospective study from May 2017 to December 2018 at a 42-bed medical ICU in a tertiary hospital with more than 1300 beds in southern Taiwan. The cases aged at least 20 years with suspected VRTIs were considered for inclusion. Similar to the real-world practice, those with positive results of microbiological tests indicative of specific causative pathogens, such as positive rapid antigen tests, blood cultures, or significant bacteria on Gram stain for endotracheal aspirates, were excluded. Written informed consents were obtained from the patients or surrogate decision-makers. The study was approved by the institutional review board of National Cheng Kung University Hospital (IRB No.: B-ER-105-350). All experiments were performed in accordance with relevant guidelines and regulations.

### Clinical data collection

Electronic medical records of included patients were reviewed for demographic variables, clinical manifestations, contact history, chronic illness, laboratory results within 48 h upon presentation, radiographic images, virological studies, causes of respiratory distress, mechanical ventilation, acute respiratory distress syndrome (ARDS), concurrent non-viral respiratory pathogens, severity scores grading by Acute Physiology and Chronic Health Evaluation II (APACHE II) on the first ICU day and Sequential Organ Failure Assessment (SOFA) score on the day of first respiratory specimen collection, length of ICU or hospital stay, duration of mechanical ventilation, ICU and 28-day mortality, and antibiotic therapy information. In addition, one of the authors (CT Cia or JC Lee) obtained symptom details from the patients or their relatives upon inclusion.

### Definitions

ARDS and its severities were defined according to the Berlin definition^17^. Vasopressor use was defined as norepinephrine, vasopressin, epinephrine, dopamine, or phenylephrine administration for 120 min or longer. Septic shock was defined as vasopressor use and hyperlactatemia without hypovolemia, based on the Sepsis-3 consensus^18^. Antibiotic-free days were defined as the calendar days without antibacterial therapy within 10 days after specimen collection for virological studies. For a patient who did not survive for 10 days, the antibiotic-free days were assigned to be zero.

### Virological studies

Nasopharyngeal swab (NPS), throat swab (TS), or bronchoalveolar lavage (BAL) from ICU patients were examined by viral cultures, real-time polymerase chain reaction (PCR) for influenza virus, and BioFire FilmArray respiratory panel (FARP) (BioFire Diagnostics, Salt Lake City, Utah, USA). The swabs were placed into in-house or commercially available viral transport medium (Copan Diagnostics, Murrieta, California, USA). Flocked swabs, instead of conventional cotton swabs, were used for the collection of nasopharyngeal or throat samples since April 2018. The decision to perform a bronchoscopy was made by the attending physicians. Other microbiological studies were ordered according to clinical needs.

Respiratory specimens in transport medium were inoculated to cell lines, including human lung carcinoma (A549), human embryonic rhabdomyosarcoma (RD), and Madin-Darby canine kidney (MDCK) cells. Tubes with cytopathic effect were confirmed by D^3^ Ultra 8™ DFA (direct fluorescent antibody) Respiratory Virus Screening and ID Kit (Diagnostic Hybrids, Athens, Ohio, USA), which was able to detect influenza A and B viruses, respiratory syncytial virus (RSV), human adenovirus (HAdv), HPIV 1–3, and human metapneumovirus (HMPV). Those without cytopathic effects were blindly stained by the same kit after inoculation for 10 days.

Nucleic acid amplification of influenza viruses was performed on LightCycler 480 real-time PCR machine (Roche Diagnostics, Rotkreuz, Switzerland) after RNA extraction with LabTurbo 48 Compact auto-extraction System (Taigen Bioscience Corp., Taipei, Taiwan). The sequences of influenza primers and probes were provided by the World Health Organization and the Taiwan Centers of Disease Control (Additional File 1, Table S1).

The FARP tests were performed by mixing 300 μL of VTM with sample buffer, injection into a test pouch containing reagents for nucleic extraction, PCR amplification, and detection of pathogen targets, including HAdv, human coronavirus (HCoV)-229E, HCoV-HKU1, HCoV-OC43, HCoV-NL63, HMPV, human rhinovirus/enterovirus (RV/EV), influenza A (A, A/H1, A/H1-2009, A/H3), influenza B, HPIV 1–4, RSV, and three bacteria (*Bordetella pertussis, Chlamydia pneumoniae,* and *Mycoplasma pneumoniae*). The pouches (version 1.7) were inserted into the FilmArray instrument (version 2.0). An equivocal result was reported as negative. Since the panel was not a routine test during the study period, the treating physician might not be informed of the results timely.

### Statistical analysis

All statistical analyses were performed using R 4.0.2 (R Foundation for Statistical Computing, http://www.R-project.org/, Vienna, Austria). Continuous variables were presented as means ± standard deviations or medians (1st–3rd quartile). Student's t test or Wilcoxon rank-sum test was applied for the between-group comparisons for normally or non-normally distributed variables, respectively. Category variables were compared by Pearson’s χ2 test or Fisher’s exact test when appropriate. The Wilson score was applied for confidence interval (CI) estimates of binomial proportions. A *p* value of less than 0.05 was considered to be statistically significant.

The multivariable logistic regression was performed to identify the factors associated with the detection of respiratory viruses. Clinical information available at the timing of samples collecting for respiratory viruses, including background information, symptoms, laboratory studies, and imaging findings, were considered for the modeling. The cases with missing data were excluded from the corresponding analyses. Of continuous variables such as age, C-reactive protein, and procalcitonin, we tried modeling with clinically relevant cut-off values using R package cutpointr, to enhance their clinical applicability. Factors with a *p* value of less than 0.15 in simple regressions were included into the multivariable model. The effect sizes were presented by odds ratios (OR) with 95% CIs.

## Results

**Figure 1:**
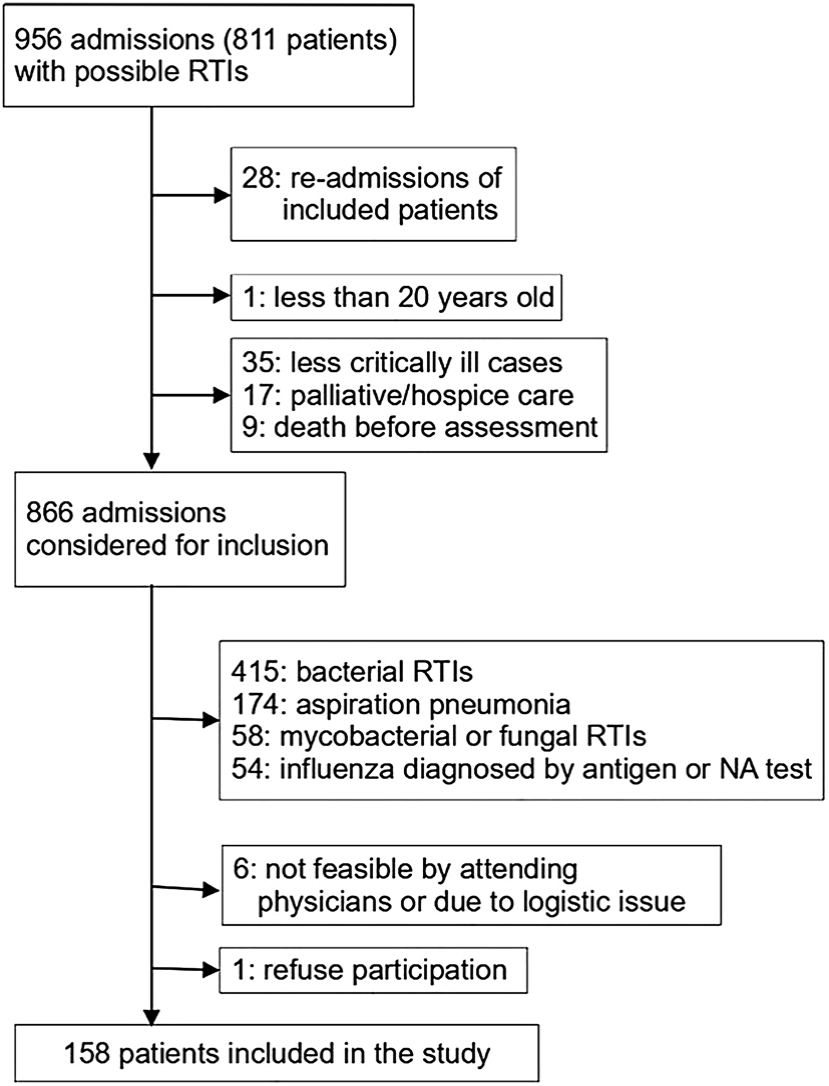
Inclusion and exclusion of the study patients. *RTIs* respiratory tract infections, *NA* nucleic acid.

Among 811 critically ill patients with 956 ICU admissions due to possible RTIs during the study period, 158 adults with 167 respiratory specimens (i.e., NPS 154, BAL 11, and TS 2 samples) were included (Fig. 1). All patients were tested by the FilmArray panel, while one and seven patients did not underwent nucleic acid amplification for influenza viruses and viral cell cultures, respectively. The first specimen collection was obtained at a median of one day (0–1 day) after ICU admission.

Respiratory viruses were detected in 64 (40.5%) patients, *i.e.,* 64 patients with VRTIs. The comparisons of clinical data between patients with or without detection of respiratory virus are demonstrated in Table 1. Patients with VRTIs were younger (median age: 63 vs. 69 years, *p* = 0.008), but there were no differences in terms of chronic illness, disease severity, and clinical outcomes, including mortality rate and length of ICU or hospital stay between the two groups.

**Table 1:** Clinical characteristics of patients with and without viral respiratory tract infections (VRTIs).

	All (n = 158)	With VRTIs (n = 64)	Without VRTIs (n = 94)	*p* value
**Demographic**				
Age	68 (57–77)	63 (50–75)	69 (60.25–79)	0.008
Male sex	87 (55%)	29 (45.3%)	58 (61.7%)	0.268
Hospital stay	16 (10–28)	16 (10–25.25)	16 (10–29.75)	0.980
**Comorbidity**				
Hypertension	72 (45.6%)	29 (45.3%)	43 (45.7%)	0.974
Diabetes mellitus	47 (29.7%)	19 (29.7%)	28 (29.8%)	0.992
Chronic kidney disease	45 (28.5%)	19 (29.7%)	24 (27.7%)	0.836
Heart failure	37 (23.4%)	16 (25.0%)	21 (22.3%)	0.761
Coronary artery disease	28 (17.7%)	14 (21.9%)	14 (14.9%)	0.348
COPD	25 (15.8%)	8 (12.5%)	17 (18.1%)	0.419
Malignancies	24 (15.2%)	8 (12.5%)	16 (17.0%)	0.503
Autoimmune diseases	11 (7.0%)	3 (4.5%)	8 (8.7%)	0.529
Bronchiectasis	8 (5.0%)	5 (7.8%)	3 (3.2%)	0.279
Asthma	4 (2.5%)	2 (3.1%)	2 (2.1%)	1.000
Hepatic cirrhosis	2 (1.3%)	0 (0%)	2 (2.1%)	0.517
**Severity and outcomes**				
Vasopressor use	64 (40.5%)	26 (40.6%)	38 (40.4%)	1.000
Septic shock	32 (20.3%)	15 (23.4%)	17 (18.1%)	0.638
Mechanical ventilation	128 (81.0%)	52 (81.3%)	76 (80.9%)	0.984
ARDS	60 (38.0%)	29 (45.3%)	31 (33.0%)	0.297
Moderate-to-severe	46 (29.1%)	21 (32.8%)	25 (26.6%)	0.533
APACHE II^a^	21.23 ± 7.66	19.98 ± 6.98	22.09 ± 8.10	0.083
SOFA score^b^	7.25 ± 3.71	7.44 ± 3.56	7.12 ± 3.81	0.590
MV days	4 (2–10)	4.5 (2–9.25)	4 (2–12)	0.892
ICU stay	8 (5–14)	8 (5–13)	8 (5–14)	0.691
Hospital stay	16 (10–28)	16 (10–25.25)	16 (10–29.75)	0.980
ICU mortality	24 (15.2%)	7 (10.9%)	17 (18.1%)	0.289
28-day mortality	23/152 (15.1%)	8/62 (12.9%)	15/90 (16.7%)	0.584

*VRTI* viral respiratory tract infection, *COPD* chronic obstructive pulmonary disease, *ARDS* acute respiratory disease syndrome, *APACHE II* Acute Physiologic Assessment and Chronic Health Evaluation II, *SOFA* Sequential Organ Failure Assessment, *MV* mechanical ventilation, *ICU* intensive care unit.

^a^On the ICU admission day.

^b^On the day obtaining the first respiratory specimen.

**Figure 2:**
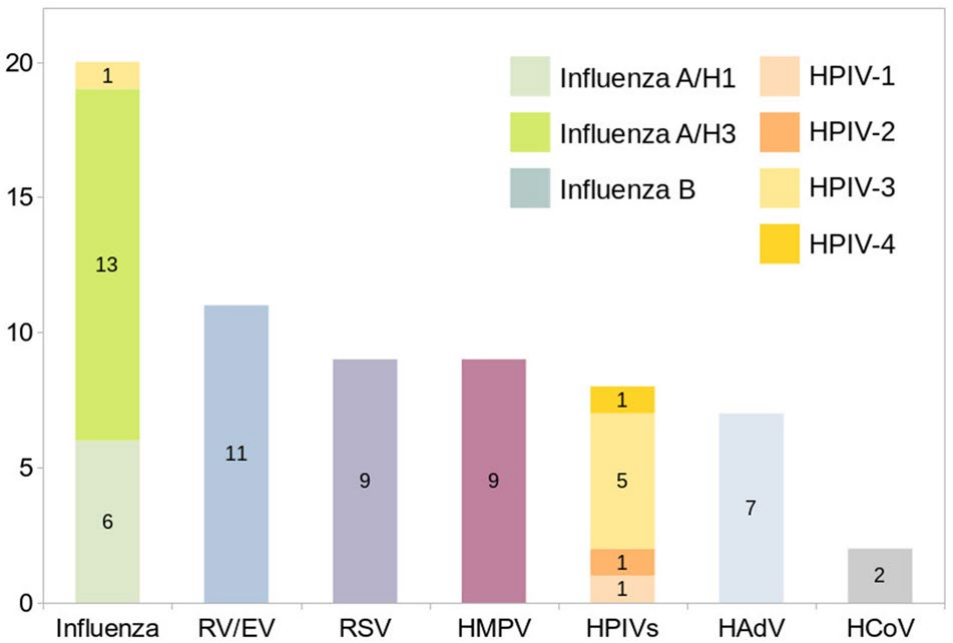
Respiratory viruses detected in 64 critically ill adults. *RV/EV* rhinovirus/enterovirus, *RSV* respiratory syncitial virus, *HMPV* human metapneumovirus, *HPIV* human parainfluenza virus, *HAdV* human adenovirus, *HCoV* human coronavirus.

The most commonly detected virus was influenza virus (20 patients: A/H1 6, A/H3 13, B 1), followed by RV/EV (11), RSV (9), HMPV (9), HPIVs (8), HAdV (7), and HCoVs (2) (Fig. 2). To be noted, 54 adults admitted to medical ICUs with confirmed influenza were not included in the study. The FARP detected respiratory viruses in 64 patients and *M. pneumoniae* in one patient. Nine patients with negative FARP results, but had VRTIs due to respiratory viruses detected by other tests, including influenza A/H3 (7), influenza A/H1 (1) and HAdV (1). Among eight patients with both NPS and BAL tested by the panel, two had discordant results: one HCoV-NL63 only in NPS and the other HAdV only in BAL. The remaining six patients had concordant NPS and BAL results, including one with influenza A/H3, one with hMPV, and four without viral pathogen. Viral cell cultures revealed respiratory viruses in 12 patients (influenza A: 3, HAdV: 3, RSV: 3, HPIV-3: 2, RV/EV: 1), accounting for 20.3% of 59 patients with VRTIs having viral cultures.

The Ct values of clinical specimens with positive results of in-house real-time PCR testing ranged from 17.4 to 35.0. Among 13 patients with influenza A/H3, the Ct values in patients with positive FARP results were significantly lower than those with negative results (26.4 ± 2.5 versus 33.2 ± 1.7, *p* < 0.001). A Ct breakpoint of 30 can well discriminate the two groups (Supplementary Fig. 1). Two FARP-negative patients with influenza A/H3 detected in the endotracheal aspirate but not upper respiratory tract specimens by in-house real-time PCR had Ct values of 32.9 and 35.0, respectively. For influenza A/H1 infection, two of the three patients with Ct values > 30 had a positive FARP results.

Twelve (18.8%) of 64 patients with and 25 (26.6%) of 94 patients without VRTIs had RTIs due to other pathogens. Of 12 patients with VRTIs, coexisting pneumonia caused by bacteria was present in nine patients, including two with *Klebsiella pneumoniae* and two with *Staphylococcus aureus* infections, while three patients had concurrent pulmonary aspergillosis. Of the latter, one had concurrent fursarosis and pneumocystis, and another biopsy-proven *Candida tropicalis* tracheobronchitis. Of co-infections due to non-respiratory viruses, one had cytomegalovirus pneumonitis and the other human simplex virus-1 tracheobronchitis. Among seven fatal patients with VRTIs (HAdV: 2, RSV: 2, RV/EV: 2, influenza A/H3: 1), two were complicated with concurrent bacterial pneumonia.

Among all 158 patients with respiratory distress, 22 (46.8%) of 47 patients with acute decompensated heart failure had VRTIs, while the proportions of VRTIs in patients with pneumonia, tracheobronchitis, acute exacerbation of chronic obstructive pulmonary disease, and asthma were 45.8% (38/83), 36.4% (4/11), 29.2% (4/24), and 50% (2/4), respectively. None of nine patients related to metabolic acidosis (5 patients), sepsis of unknown source (3), or *K. pneumoniae* bacteremia complicated by liver abscess (1) had VRTI.

**Figure 3:**
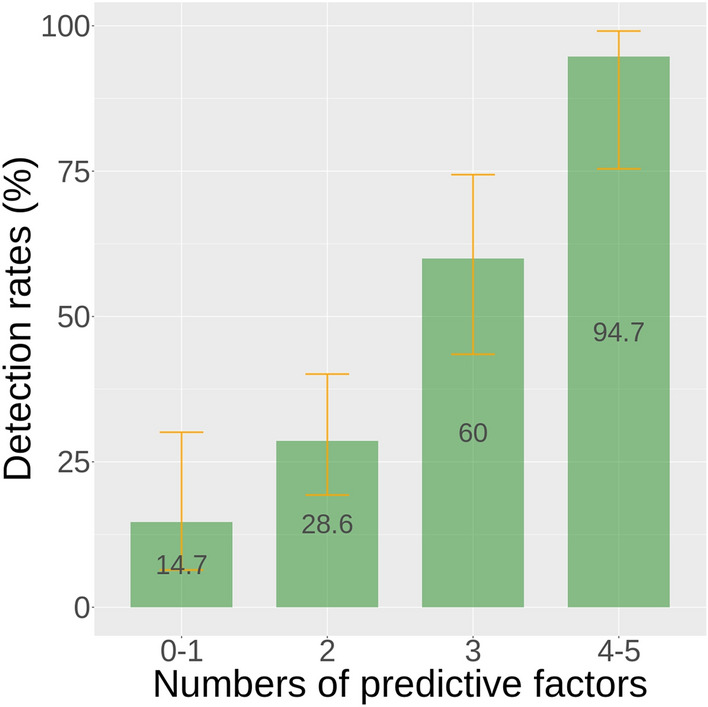
Numbers of predictive factors and detection rates of respiratory viruses. Error bars represent the 95% Wilson score confidence intervals of the.

The multivariable logistic regression analyses revealed the risk factors associated with VRTIs in Table 2. The variables significantly associated with VRTIs in the simple regression analysis but not in the multivariable model included myalgia, rhinorrhea, neutrophil-to-lymphocyte ratio > 12.5, and specimen collected by a flock swab. None of the blood biomarkers, such as white blood cell count, serum levels of C-reactive protein or procalcitonin, were predictive of the existence of VRTIs. Age less than 65 years old (OR, 4.61; 95% CI, 1.99–11.40), household contact with someone having upper RTI (OR, 4.28; 95% CI 1.82–10.82), the presence of fever (OR, 3.16; 95% CI 1.23–8.81), productive cough (OR, 3.42; 95% CI, 1.45–8.63), or sore throat (OR, 3.82; 95% CI 1.27–12.59), were significantly associated with VRTIs. The association of the number of the above variables with probability of VRTI was shown in Fig. 3, Supplementary Fig. 1, and Table S2.

**Table 2:** Simple and multivariable regressions for factors associated with viral respiratory tract infections.

Variables	Simple regression		Multiple regressions	
	Odds ratio (95% CI)	*p* value	Odds ratio (95% CI)	*p* value
Age < 65 years	3.03 (1.57–5.94)	0.001	3.98 (1.72–9.65)	0.002
Household contact with a person having upper RTI	2.76 (1.42–5.45)	0.003	3.93 (1.66–9.86)	0.002
**Clinical symptoms**				
Rhinorrhea	3.70 (1.87–7.47)	< 0.001	2.23 (0.90–5.67)	0.085
Sore throat	3.68 (1.65–8.60)	0.002	3.70 (1.24–11.98)	0.022
Myalgia	2.95 (1.30–6.97)	0.011	2.06 (0.76–5.79)	0.159
Productive cough	2.87 (1.18–7.56)^a^	0.024	3.24 (1.38–8.12)^b^	0.009
Fever	2.29 (1.05–5.34)	0.044	2.89 (1.12–8.00)	0.032
Dyspnea	2.11 (0.47–14.75)	0.369		
Dry cough	1.48 (0.55–4.21)^a^	0.442		
Headache	1.35 (0.48–3.73)	0.561		
Malaise	1.14 (0.60–2.18)	0.678		
Altered mental status	0.64 (0.24–1.56)	0.347		
**Laboratory tests**				
White blood cell count	1.00 (1.00–1.00)	0.513		
Leukocytosis^c^	0.75 (0.40–1.43)	0.389		
Leukopenia^d^	1.10 (0.21–5.19)	0.897		
NLR	1.01 (0.99–1.04)	0.231		
NLR > 12.5	1.79 (0.90–3.55)	0.094	2.28 (0.94–5.73)	0.073
C-reactive protein (CRP)^e^	1.00 (0.99–1.01)	0.849		
CRP ≥ 89 mg/L	2.14 (0.44–11.31)	0.348		
Procalcitonin (PCT)^f^	1.01 (0.97–1.06)	0.601		
PCT > 0.35 ng/mL	1.63 (0.58–4.62)	0.355		
**Patterns on chest film**^**g**^				
No new lung lesion	0.38 (0.13–0.96)	0.054	0.70 (0.21–2.14)	0.542
Multifocal consolidations	1.24 (0.66–2.36)	0.503		
Bilateral GGOs	1.05 (0.55–1.98)	0.897		
Bilateral reticular pattern	1.06 (0.21–5.19)	0.882		
Pulmonary edema pattern	1.53 (0.80–2.94)	0.198		
Pleural effusion	0.70 (0.35–1.36)	0.298		
Flocked swab use	1.99 (1.04–3.84)	0.038	2.11 (0.95–4.84)	0.070

*CI* confidence interval, *RTI* respiratory infection, *NLR* neutrophil-to-lymphocyte ratio.

^a^Compared to no cough.

^b^Compared to no or dry cough.

^c^White blood cell count > 11,000 /μL.

^d^White blood cell count < 4,000 /μL.

^e^Available in 27 patients.

^f^Available in 62 patients.

^g^Patterns not undergoing modeling due to limited patient numbers: unilobar consolidation (4), pneumothorax (2), mass or cavity (0) point estimates.

For antibiotic exposure, all patients received antibacterial agents on the first date of respiratory specimen collection for virological studies. The antibiotic-free days within 10 days were similar between patients with and without VRTIs (median 0 [0–3.5] versus 0 [0–2.75] days, *p* = 0.15). However, those with VRTIs had more calendar days free from intravenous antibiotics (median 4 [1–6] versus 2 [0–5.75] days, *p* = 0.07), though the difference was not statistically significant.

## Discussion

The current study assessed the prevalence of VRTIs in pragmatically selected adults with critical illness, when other causes were unable to fully explain the patients’ respiratory distress and clinical presentation. A positivity rate of 40.5% in the present study is higher than that of non-selected patients receiving mechanical ventilation in ICUs^5^. The median time from ICU admission to specimen collection for multiplex test is one day, indicating that the tests were ordered according to clinical information obtained right after ICU admission, rather than reserving the tests for patients with negative results of bacterial cultures.

To our knowledge, the host variables predictive of VRTIs among critically ill patients had not been adequately investigated before, as previous studies included mainly demographic information, comorbidities, or laboratory results^7–9^. Age < 65 years old, household contact with an individual having upper RTI and the presence of fever, productive cough, or sore throat were linked to VRTIs. In the patients presenting three of the above five characters, the probability of VRTIs is 60%. Despite the difficulty to obtain detail information from those with invasive mechanical ventilation or with altered mental status, which accounted for 81% or 16% of the included patients respectively, the associations between clinical symptoms and VRTIs remained statistically significant, while most laboratory tests or chest X-ray findings failed to predict the presence of VRTIs. The indispensability of thorough history taking should be emphasized in critical care settings. The age factor could be attributed to a higher rate of non-infectious causes of respiratory distress in the elderly^19,20^. It is not surprising that traditional laboratory tests failed to detect respiratory viruses in clinical samples, since bacterial etiological pathogens could be noted in only 13.2% of the included patients.

Despite there were 57 cases of severe influenza not included in this study, influenza virus remained to be the most commonly viral pathogen. The weighting of influenza virus is obviously underestimated in the viral etiologies of RTIs. Thus, our prevalence data supports empiric administration of anti-influenza agents to selected critically ill patients with suspected VRTIs. However, the FARP failed to detect influenza A/H3 in 7 (53.8%) out of the 13 patients diagnosed by the in-house PCR using updated primers as these patients had relatively lower viral loads reflected by higher Ct values. As influenza viruses evolve rapidly, physicians should be cautious that commercially available molecular assays may provide unreliable results, if not updated frequently^21^.

As influenza virus, other respiratory viruses can cause significant morbidity and mortality in critically ill patients^22,23^, and in this study these viruses accounted for 68.8% of all VRTIs. Without the use of multiplex respiratory virus testing, the prevalence of non-influenza VRTIs would be frankly underestimated, as only 22% (9/41) patients with non-influenza VRTIs had virus isolated in the current study. To detect these respiratory viruses, previous studies have reported clinically acceptable sensitivities of the FARP test, 80% to100%, except for adenoviruses^24–26^. Six of seven patients died in the ICUs were infected by non-influenza viruses. However, our case number is insufficient to compare clinical characteristics among different respiratory viruses and further studies are warranted.

Previous studies showed that clinical specimens from upper and lower respiratory tract specimens may yield discordant testing results, as noted in 2 (25%) of our 8 patients with both NPS and BAL samples tested for respiratory viral pathogens, in 11 to 36% of critically ill or immunosuppressed hosts^5,6,27,28^. Accordingly, etiological surveys using both upper and lower respiratory tract specimens ought to be considered in patients with highly suspected VRTIs.

Clinical application of multiplex respiratory viral assays had been expected to facilitate discontinuation or de-escalation of antibiotics^29^. Interestingly, several studies with the detection rates of < 25% for respiratory viruses invariably show no significant decline in antibiotic use^30–32^. When the prevalence of VRTIs exceeded 25%, the utilization of these assays was associated with favorable results on antimicrobial stewardship program (Supplementary Table S3)^30–37^. None of these studies focused on ICU patients. In the current study, all patients received antibiotics and those with VRTIs tended to receive less intravenous antibiotics, indicating that the intensivists may view these virological testing results as supportive evidence for antibiotic de-escalation. Our detection rate of 40.5% for viral pathogens suggested that further investigations for the impact of multiplex viral testing on antimicrobial stewardship in critically ill patients are worth expecting.

Our study inherited several limitations. First, this is a single-center observation study and the study result cannot be generally applied to other clinical settings or healthcare facilities, since the distribution of respiratory viruses varies seasonally and geographically. The enrollment of the study participants reflected the discretion of attending physicians to consider VRTIs among critically ill patients, while universal testing or other strategies may yield different results. Second, only 7% of our included patients had lower respiratory tract specimen tested, and thus the rate of VRTIs are likely to be underestimated^5,6^. Third, the case of concurrent viral and non-viral RTIs were largely excluded since according to the study protocol, the initial clinical presentation would preclude respiratory virus tests. Fourth, a significant proportion of patients with influenza were excluded due to positive influenza results at the time of screening and the weight of influenza was likely to be decreased, so the clinical characters associated with VRTIs may be varied, if all cases of VRTIs were included for the analysis.

## Conclusions

VRTIs were not uncommon in pragmatically selected adults with critical illness. Age < 65 years old, household contact with a person having upper RTI, and the presence of clinical symptoms of fever, productive cough, or sore throat, were associated VRTIs. To maximize clinical benefit, intensivists can consider selective application of multiplex molecular assays for respiratory viral pathogens, based on the above predictive variables. The sensitivity and specificity of the predicative model and clinical impact of VRTIs need to be further studied on larger prospective cohorts.

## Supplementary Information

Supplementary Information.

## Data Availability

The datasets generated during and/or analysed during the current study are available from the corresponding author on reasonable request.

## Abbreviations

RTI: Respiratory tract infection
ICU: Intensive care unit
HCoV: Human coronavirus
VRTI: Viral respiratory tract infection
ARDS: Acute respiratory distress syndrome
APACHE II: Acute Physiology and Chronic Health Evaluation II
SOFA: Sequential Organ Failure Assessment
NPS: Nasopharyngeal swab
TS: Throat swab
BAL: Bronchoaveolar lavage
PCR: Polymerase chain reaction
RARP: BioFire FilmArray respiratory panel
VTM: Viral transport medium
RD: Rhabdomyosarcoma
MDCK: Madin-Darby canine kidney
RSV: Respiratory syncytial virus
HAdv: Human adenovirus
HPIV: Human parainfluenza virus
HMPV: Human metapneumovirus
RV/EV: Human rhinovirus/enterovirus
OR: Odds ratio
