# Supplementary material for: Respiratory viral infections in pragmatically selected adults in intensive care units
Source: Sci Rep. 2021 Oct 8;11:20058. doi: 10.1038/s41598-021-99608-y (PMC8501073; doi:10.1038/s41598-021-99608-y)
Supplement: Supplementary file 1 — Supplementary Information. [file 41598_2021_99608_MOESM1_ESM.doc]

**Table S1.** Primers and probes for in-house amplification of influenza viral nucleic acid

| Primers or probes | Sequences |
| --- | --- |
| IA-F | 5’-GACCRATCCTGTCACCTCTGAC-3’ |
| IA-R | 5’-AGGGCATTYTGGACAAAKCGTCTA-3’ |
| IA-probe | 5’-FAM-TGCAGTCCTCGCTCACTGGGCACG-3’-BHQ1 |
| N-IB-F | 5’-GAGACACAATTGCCTACCTGC TT-3’ |
| N-IB-R | 5’-TTCTTTCCCACCGAACCAAC-3’ |
| N-IB probe | 5’-FAM-AGAAGATGGAGAAGGCAAAGCAGAACTAGC-3’-BHQ1 |
| SW H1-F | 5’-GCACGGTCAGCACTTATYCTRAG-3’ |
| SW H1-R | 5’-GTGRGCTGGGTTTTCATTTGGTC-3’ |
| SW H1 probe | 5’-FAM-CYACTGCAAGCCCA”T”ACACACAAGCAGGCA-3’‐BHQ1^a^ |
| H3-266-F (mutant) | 5’-ACCCTCAGTGTGATGGCTTTCAAA-3’ |
| H3-373-R (mutant) | 5’-TAAGGGAGGCATAATCCGGCACAT-3’ |
| H3-315 probe | 5’-FAM-ACGAAGCAAAGCCTACAGCAACTGTT-3’-BHQ1 |

^a^ Taqman® probes are labeled at the 5'-end with the reporter molecule 6-carboxyfluorescein (FAM) and quenched internally at a modified “T” residue with BHQ1, with a modified 3’-end to prevent probe extension by Taq polymerase.

**Table S2.** Numbers of predictive factors and detection rates of respiratory viruses

| Numbers of predictive factors ^a^ | Detection rate (patient number) | 95% confidence interval ^b^ |
| --- | --- | --- |
| 0–1 | 12.1% (4/33) | 4.8%–27.3% |
| 2 | 29.6% (21/71) | 20.2%–41.0% |
| 3 | 60.0% (21/35) | 43.5%–74.4% |
| 4–5 | 94.7% (18/19) | 75.4%–99.1% |

^a^ five predictive factors: age < 65 years, household contact with individuals with upper respiratory tract infection, fever, productive cough, sore throat.

^b^ derived from Wilson score.

Note: Five patients without predictive factors had no virus detected, and 3 patients with five factors had viral respiratory tract infections.

**Figure S1**. Cycle threshold (Ct) values of real-time polymerase chain reaction for influenza A/H3 and the FilmArray® Respiratory Panel (FARP) results


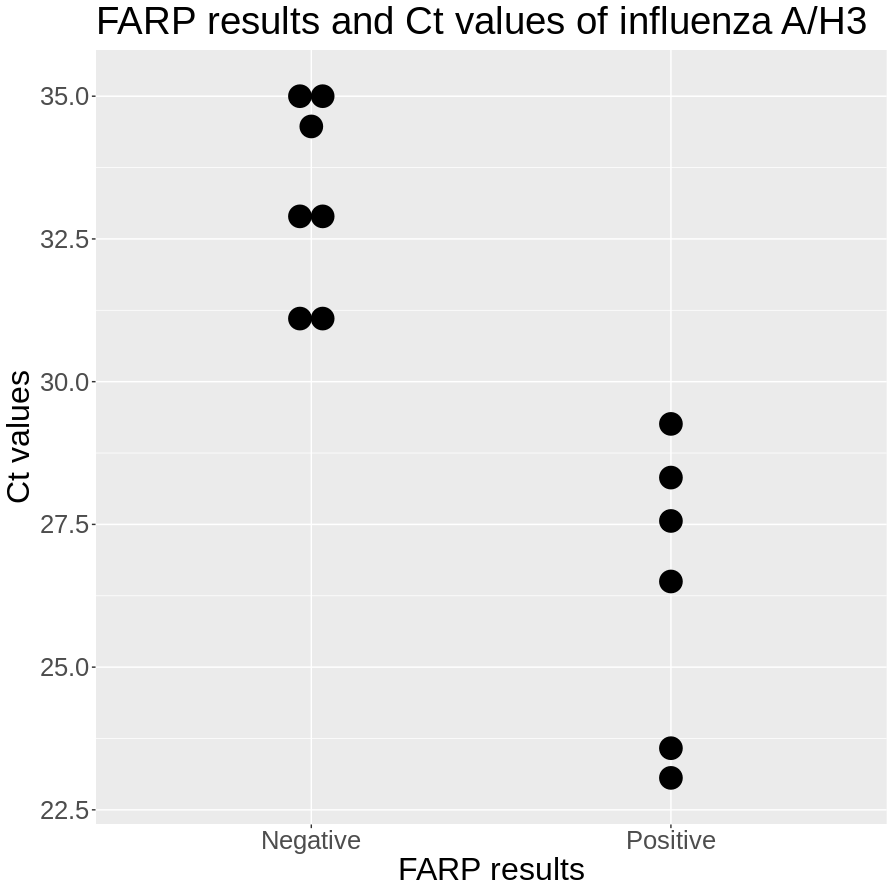


**Table S3.** Randomized or quasi-randomized trials investigating the impact of multiplex viral assays on antibiotics use

| Authors,  published year | Country | Design | Settings | Prevalence of RV infection | Impact on antibiotics use | Reference |
| --- | --- | --- | --- | --- | --- | --- |
| Oosterheert et al,  2005 | Netherlands | Randomized controlled trial | Adult inpatients with lower RTIs | 22.4% (24/107) | No reduction | [31] |
| Andrews et al,  2017 | UK | Quasi-randomized trial | Adult inpatients and outpatients with upper +/- lower RTIs | 22.8% (124/545) | No reduction | [32] |
| Saarela et al,  2019 | Finland | Randomized controlled trial | Adult ED patients with respiratory symptoms, fever, chest pain, or poor general condition | 17.5% (175/998) | No reduction | [30] |
| Branche et al,  2015 | USA | Randomized controlled trial | Adult inpatients with non-pneumonic lower RTIs. Procalcitonin-guided antibiotic algorithm. | 42.7% (128/300) | Decreased antibiotics at discharge in patients with positive RV and low procalcitonin | [34] |
| Brendish et al,  2017 | UK | Randomized controlled trial | Adult patients at ED or acute medical unit with acute respiratory illness or fever | 44.7% (161/360) | More frequent single dose or short course antibiotics in the RV testing group. | [35] |
| Echayarria et al  2018 | Argentina | Randomized controlled trial | Adult and pediatric ED patients with lower RTIs | 83.7% (242/289) | Reduced antibiotic prescriptions | [37] |
| Shengchen et al, 2019 | China | Randomized controlled trial | Adult inpatients with lower RTIs | 28.9% (115/398) | Shorter antibiotic duration, more frequent de-escalation | [33] |
| May et al,  2019 | USA | Randomized controlled trial | Adult and pediatric ED patients with upper RTIs or influenza-like illness | 57.0% (53/93) | A trend toward decreased antibiotic use (–12%; 95% CI, 25%-0.4%; P = 0.06/0.08) | [36] |

*ED* emergency department, *RTI* respiratory tract infection, *RV* respiratory virus, *CI* confidence interval
